# Repositioning drugs by targeting network modules: a Parkinson’s disease case study

**DOI:** 10.1186/s12859-017-1889-0

**Published:** 2017-12-28

**Authors:** Zongliang Yue, Itika Arora, Eric Y. Zhang, Vincent Laufer, S. Louis Bridges, Jake Y. Chen

**Affiliations:** 1 Center for Biomedical Big Data, Wenzhou Medical University First Affiliated Hospital, Wenzhou, Zhejiang Province China; 2 Informatics Institute in School of Medicine, University of Alabama at Birmingham, Birmingham, 35233 AL USA; 3 Division of Clinical Immunology and Rheumatology in School of Medicine, University of Alabama at Birmingham, Birmingham, 35233 AL USA; 4 Wenzhou Yuekang InfoTech, Ltd., Wenzhou, Zhejiang Province China

## Abstract

**Background:**

Much effort has been devoted to the discovery of specific mechanisms between drugs and single targets to date. However, as biological systems maintain homeostasis at the level of functional networks robustly controlling the internal environment, such networks commonly contain multiple redundant mechanisms designed to counteract loss or perturbation of a single member of the network. As such, investigation of therapeutics that target dysregulated pathways or processes, rather than single targets, may identify agents that function at a level of the biological organization more relevant to the pathology of complex diseases such as Parkinson’s Disease (PD). Genome-wide association studies (GWAS) in PD have identified common variants underlying disease susceptibility, while gene expression microarray data provide genome-wide transcriptional profiles. These genomic studies can illustrate upstream perturbations causing the dysfunction in signaling pathways and downstream biochemical mechanisms leading to the PD phenotype. We hypothesize that drugs acting at the level of a gene expression module specific to PD can overcome the lack of efficacy associated with targeting a single gene in polygenic diseases. Thus, this approach represents a promising new direction for module-based drug discovery in human diseases such as PD.

**Results:**

We built a framework that integrates GWAS data with gene co-expression modules from tissues representing three brain regions—the frontal gyrus, the lateral substantia, and the medial substantia in PD patients. Using weighted gene correlation network analysis (WGCNA) software package in R, we conducted enrichment analysis of data from a GWAS of PD. This led to the identification of two over-represented PD-specific gene co-expression network modules: the **Br**own Module (**Br**) containing 449 genes and the **T**urquoise module (**T**) containing 905 genes. Further enrichment analysis identified four functional pathways within the Br module (cellular respiration, intracellular transport, energy coupled proton transport against the electrochemical gradient, and microtubule-based movement), and one functional pathway within the T module (M-phase). Next, we utilized drug-protein regulatory relationship databases (DMAP) and developed a **D**rug **E**ffect **S**um **S**core (***DESS***) to evaluate all candidate drugs that might restore gene expression to normal level across the Br and T modules. Among the drugs with the 12 highest *DESS* scores, 5 had been reported as potential treatments for PD and 6 hold potential repositioning applications.

**Conclusion:**

In this study, we present a systems pharmacology framework which draws on genetic data from GWAS and gene expression microarray data to reposition drugs for PD. Our innovative approach integrates gene co-expression modules with biomolecular interaction network analysis to identify network modules critical to the PD pathway and disease mechanism. We quantify the positive effects of drugs in a *DESS* score that is based on known drug-target activity profiles. Our results illustrate that this modular approach is promising for repositioning drugs for use in polygenic diseases such as PD, and is capable of addressing challenges of the hindered gene target in drug repositioning approaches to date.

**Electronic supplementary material:**

The online version of this article (10.1186/s12859-017-1889-0) contains supplementary material, which is available to authorized users.

## Background

Parkinson’s Disease (PD) is a disorder characterized by depletion of dopamine in the basal ganglia, including the substantia nigra. While the exact etiology of PD is unknown, major advances have been made in understanding underlying disease mechanisms through technologies in genetics, transcriptomics, epigenetics, proteomics and imaging [1]. These advances have increased recognition of the heterogeneity and etiological complexity of PD as a disease. Nevertheless, there is hope for broad-spectrum therapeutic intervention, as even distinct disease subtypes implicate genes intersecting in common pathways [2]. Recently described “Network Medicine” [3] approaches offer a platform to study the molecular complexity of a particular disease systematically. These approaches are well-suited to the identification of disease modules and pathways as well as the molecular relationships between apparently distinct phenotypes [4]. Despite progress towards the understanding of genetic factors that contribute to the etiology of PD, current treatments are aimed at clinically apparent PD — after patients are suffering from the onset of neurodegeneration. While, preventative drugs aim at treatment before or during the pre-clinical stage of PD are lacking, as are curative drugs aimed at the underlying molecular mechanisms have had limited success [5].

The associations discovered in GWAS of PD allow for the identification of disease-specific modules playing a role in triggering the disease. Similarly, gene expression microarray data provides a gross overview of gene expression changes that are associated with diseases like PD. However, future studies of complex diseases will need to move beyond the analysis of single genes and include analysis of interactions between genes or proteins, in order to better understand how functional pathways and networks become dysfunctional [6]. For instance, network-based approaches have already been used to examine various disease molecular mechanisms, e.g., type-2 diabetes [7], cancer [8], and neuronal degeneration specifically [9]. Bioinformatics techniques to characterize network topology and functional modules have been developed recently for functional genomics [10]. The identification of disease modules involving specific mutated genes and the molecular pathways to which they belong will provide new targets for drug development. GWAS and whole exome profiling data are combined in systems biology to illustrate upstream perturbations causing dysfunction in pathways and mechanisms leading to the disease phenotype. Therefore, we introduce the approach of discovering disease-specific modules to reveal the etiology of PD.

In this study, we hypothesize that study of PD GWAS [11] and co-expression data [12] will enable identification of disease-specific modules caused by a variation in multiple components of a functional pathway or network. Thus, we propose using a network-based approach called Weighted Gene Co-expression Network Analysis (WGCNA) [13] to detect modules of co-expressed gene networks associated with PD. We then integrate these co-expression clusters with gene regulatory network information and perform enrichment analysis to find PD-specific modules. This method, in combination with functional enrichment and network topology measures, will be used to identify potential targets. This is done by selecting drugs that reverse the altered gene expression signatures found within the PD modules.

PD modules which show significant perturbation is identified by comparing global co-expression networks in PD to regulatory networks identified using GWAS 'hits'. After selecting the PD-specific modules for further analysis, we find significantly enriched Kyoto Encyclopedia of Genes and Genomes (KEGG) pathways and Gene Ontology terms associated with PD modules. Afterward, we use knowledge of these functional pathways as the basis for “modular drug discovery”—the discovery of drugs that act on many nodes within the disease-specific module. This is accomplished through our innovative **D**rug **E**ffect **S**um **S**core (***DESS***) system and then cross-validated through rigorous analysis of published literature.

## Methods

### An overview of the framework

**Fig. 1:**
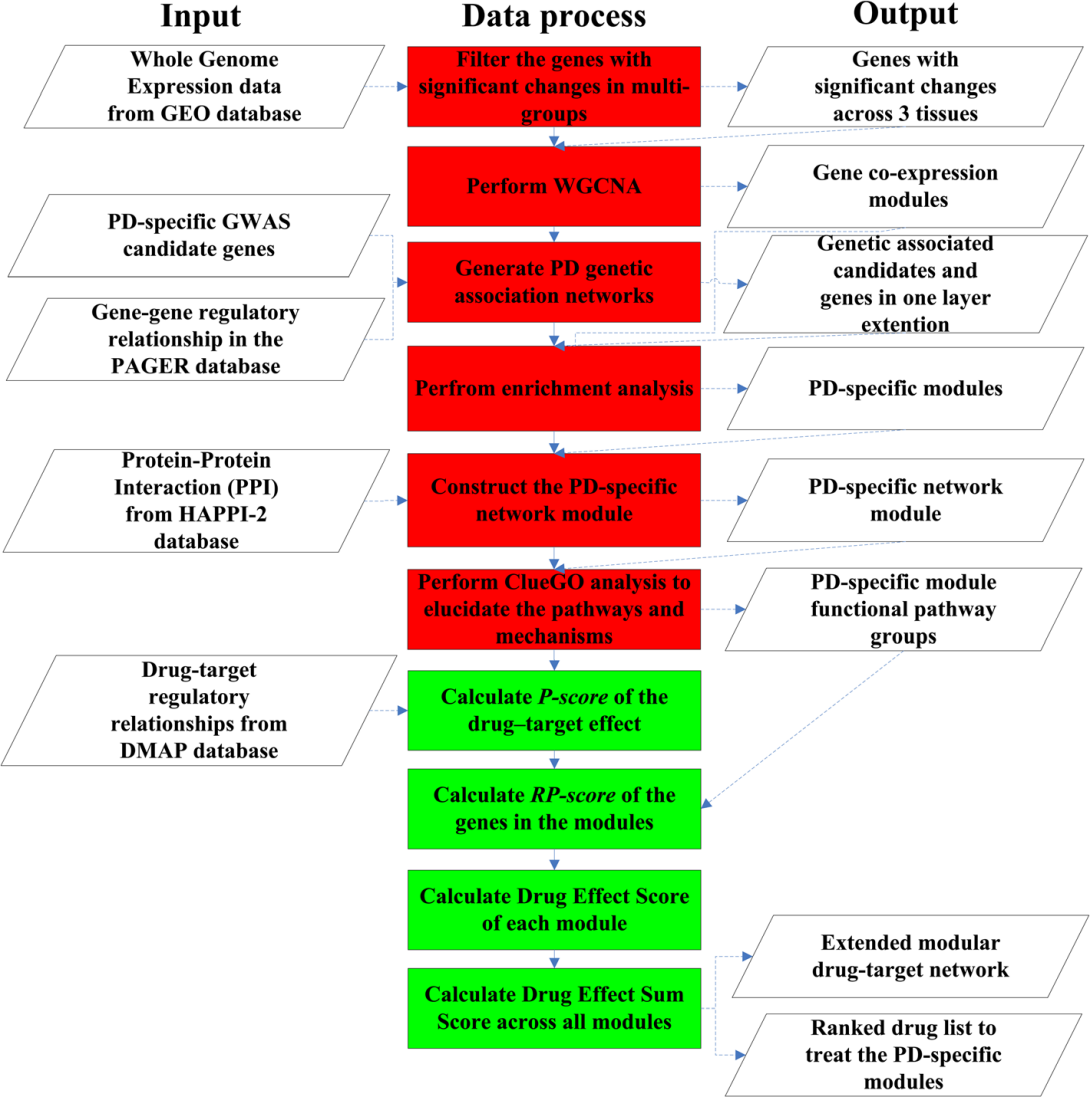
The pipeline for mining the PD-specific gene modules and for ranking candidate drugs for drug repositioning. The left frames are the source of the input data, the middle frames are the processes of data, and the left frames are the output of the process. The red frames relate to mining PD-specific modules, while those in green relate to the drug repositioning process

The pipeline is divided into two color-coded sections as shown in Fig. 1. The first section (colored red) contains steps for construction of PD modules, and the second section (colored green) contains steps to perform modular drug repositioning. The construction of PD modules was carried out in 6 steps: 1) We filtered genes with significant expression changes between the case vs. control samples (with the False Discovery Rate set to 0.05), using a Bayesian inference technique available in the limma package in R [14]. 2). We performed WGCNA, which yields clusters (modules) of highly correlated genes having significant changes across three tissues. 3) We compiled PD-specific GWAS candidate genes and performed one layer extension to generate a gene regulatory network by retrieving the gene-gene regulatory relationship from the PAGER database [15]. 4) We performed enrichment analysis by finding overlapping genes shared between co-expression clusters and GWAS candidate genes, extracting these enriched clusters as PD-specific modules. 5) We constructed PD-specific network module by retrieving the gene-gene interactions for the genes in PD-specific modules from the HAPPI-2 database [16]. 6) Finally, we annotated PD-specific modules with functional groups using ClueGO [17].

The Drug repositioning section (green) was comprised of four steps. First, we calculated a *P-score*, which is an intuitive pharmacology score that combines the probability for each interaction and the weight of the drug-target interaction using data from the DMAP database (see details in Methods). Second, we calculated the *RP-score*, which is a measure of Relevant Protein importance in the PD modules network (see details in Methods). Third, we calculated the Drug Effect Score (*DES*) of each module. Finally, the *DESS* was calculated across all modules. Using these steps, we obtained a ranked “modular drug list” consisting of candidate treatments based on PD-specific modules.

### Preparation of PD-specific omics, gene-gene interaction, and drug-protein regulation data

Datasets from whole genome expression transcriptional profiling (on the GSE8397-GPL-96 array) were retrieved from the Gene Expression Omnibus (GEO) database (https://www.ncbi.nlm.nih.gov/geo/query/acc.cgi?acc=GSE8397). In the gene expression profile, 47 samples from PD patients and controls were used in three brain regions: the Frontal Gyrus (FG: 8 tissue samples), Lateral Substantia (LS: 16 tissue samples) and Medial Substantia (MS: 23 tissue samples) [12]. SNP data was obtained from a PD paper [11], in which a GWAS was carried out. We mapped probe IDs to gene symbols using the NCBI microarray toolkit and assigned gene expression scores by the averaging probe expression values after adjustment and trimming of background noises by using the standard deviation of the mean values from all samples. Since the standard deviation of the mean values were small enough (0.02 in this study), no samples had been trimmed. After performing probe transformation and synonymous gene merging on data from the Affymetrix Human Genome U133A Array [HG-U133A] and Affymetrix Human Genome U133B Array [HG-U133B], 12,995 genes were mapped by 22,283 probes in the merged matrix from the two arrays. In the prior study, 54 genes were reported as having had significant enrichment [11] in GWAS. The PAGER database [15] was used to obtain gene-gene regulatory relationships (22,127 pairs curated from 645,385 in total). The HAPPI-2 database [16] was used to obtain protein-protein interaction (PPI) data. This integrated protein interaction database comprehensively integrates weighted human protein-protein interaction data from a wide variety of protein-protein database sources. After mapping the proteins to genes using UniProt IDs, we obtained 2,658,799 gene-gene interactions. The drug-target regulatory relationships data was from the DMAP database [18], which consisted of curated 438,004 drug-protein regulatory relationships.

### PD-specific network module identifications

Whole-genome expression data on 12,995 genes was filtered down to 2895 candidate genes, based on a multi-group empirical Bayesian (eBayes) moderated t-test with *p-value* ≤ 0.05. Next, we performed WGCNA to cluster these genes based on their co-expression. To do this, we first performed our pipeline steps to identify excessive missing values and outlier microarray samples. The detection of the outlier was performed by trimming the hierarchy tree of average Euclidean distance method using cutoff tree height of 100. Second, we chose an exponent for soft thresholding based on analysis of network topology, to further reduce noise and amplify stronger connections in the scale-free topological model. Third, we performed one-step network construction and module detection using hierarchy tree of unsigned TOM-based dissimilarity distance. Fourth, we visualized the genes in modules in a hierarchy tree based on average linkage clustering [13]. Fifth, we analyzed the cluster (Principal Components) and sample (expression data) correlation using Pearson correlation and asymptotic *p-value*.

An initial regulatory relational network was seeded using the 54 candidate genes identified by Moran et al. and expanded using the gene-gene regulatory relationship data. The resulting expanded regulatory relational network consists of 288 genes and 1983 gene-gene regulatory relationships. Subsequently, we performed the enrichment testing of the genes in the expanded regulatory relational network to measure enrichment in the co-expression clusters using the hypergeometric test and assigned the *f(pts)* score using the formula:

1 $$ \mathrm{f}\left(\mathrm{pts}\right)=\mathsf{\operatorname{sign}}\left(\frac{\mathrm{K}}{\mathrm{N}}-\frac{\mathrm{k}}{\mathrm{n}}\right)\times \boldsymbol{\mathsf{\log}}\left(\frac{\left(\genfrac{}{}{0pt}{}{\mathrm{K}}{\mathrm{k}}\right)\left(\genfrac{}{}{0pt}{}{\mathrm{N}-\mathrm{K}}{\mathrm{n}-\mathrm{k}}\right)}{\left(\genfrac{}{}{0pt}{}{\mathrm{N}}{\mathrm{n}}\right)}\right) $$

where *N* is the total number of the genes in co-expression clusters, *K* is the number of overlapping genes between co-expression clusters and genetic candidate genes, *n* is the number of the genes in the co-expression cluster selected, and *k* is the overlap genes between selected co-expression cluster and the genetic candidate genes. A positive value for *f(pts)* indicates the over-representation in the expanded regulatory relational network. PD-specific modules were defined as the over-represented co-expression clusters. We then generated the network of PD-specific modules by applying high-confidence gene-gene interactions (as indicated by 3-star or above in the HAPPI-2 database).

In the final step, we performed ClueGO analysis to elucidate mechanisms involved in the PD-specific modules. We applied Bonferroni correction and selected those with post-correction *p-value* ≤ 0.05 and *Kappa score* ≥ 0.5 (moderate network strength or stronger) [17].

### Modular drug repositioning

*DESS* was calculated using the *P-score* from the DMAP database, the *RP-score* from the PD modules, and the module enrichment score *f(pts)* from the PD modules. We calculated a *P-score*, an intuitive pharmacology score in the DMAP database, via a probability-weighted summary of all the evidence mined from literature or other drug target databases to determine an overall mechanism of “edge action” for each specific chemical-protein interaction using *conf(d,p)*:

2 $$ \mathsf{conf}\left(d,p\right)={\sum}_{i=1}^N\left({prob}_i\left(d,p\right)\times {\operatorname{sign}}_i\right) $$

where *d* and *p* are specific drugs and proteins, respectively. *N* is the number of types of evidence for the interaction between *d* and *p*. *prob*_*i*_(*d*, *p*) is confidence in each type of evidence *i* with a value within the range of [0,1]. sign_*i*_ has a value of 1 if the evidence *i* suggests activation and a value of −1 if the evidence *i* suggests inhibition. Afterwards, to rank each interaction, we used the algorithm in HAPPI [19] by assigning a *weight(p)* for the proteins interacting with each drug using the following formula adapted from [20].

3 $$ weight(p)=k\times \mathit{\ln}\left({\sum}_{p,q\in NET} conf\left(p,q\right)\right)-\mathit{\ln}\left({\sum}_{p,q\in NET}N\left(p,q\right)\right) $$

Here, *p* and *q* are proteins in the protein interaction network, *k* is an empirical constant (*k* = 2 in this study), *conf(p,q)* is the confidence score of interaction between protein *p* and *q* assigned by HAPPI-2, and *N(p, q)* holds the value of 1 if protein *p* interacts with *q* or the value of 0 if protein *p* does not interact with *q*. Thus, the foregoing probabilities and weights for each interaction were combined into *P-score(d,p)*, which includes both information on each drug’s effects on interacting proteins and the importance of the protein in the protein-protein interaction network:

4 $$ P- score\left(d,p\right)= conf\left(p,q\right)\times weight(p) $$

We applied each gene’s *RP-score* calculation in a manner similar to formula (3) in PD-specific modules using the formula:

5 $$ RP- score={e}^{k\times \ln \left({\sum}_{p,q\in ModuleNET} conf\left(p,q\right)\right)-\ln \left({\sum}_{p,q\in ModuleNET}N\left(p,q\right)\right)} $$

where *p* and *q* are the indexes of proteins from the selected module, *k* is a constant (*k* = 2 in this study). The term *conf(p, q)* is the interaction confidence score assigned by HAPPI-2, where *conf(p, q)* **∈** [0,1].

Further, we calculated a *DES(d,m)* by using the drug weight score and the module gene *RP-score* according to the formula:

6 $$ DES\left(d,m\right)={\sum}_{i\in ModT\arg et}^n\left[{\operatorname{sign}}_d\times {\log}_2\left(p-\mathrm{score}\left(d,i\right)\right)\times {\log}_2\left( RP-\mathrm{score}\left(d,i\right)\right)\times {p}_i\right] $$

where *m* is the module, *i* is the index of the proteins in the PD-specific module, sign_*d*_ is the direction of the effect drug *d* on protein expression, and *P-score(d, i)* is the pharmacology score of the drug *d* to target *i*. *p*_*i*_ is the priority score which indicates the source of the candidate. We assigned a value of *p*_i_=1/2^0^ when the candidates were from GWAS, *p*_i_=1/2^1^ when the candidates were from regulatory one-layer extension of GWAS, and *p*_i_=1/2^2^ when the other candidates were from the same module.

**Fig. 2:**
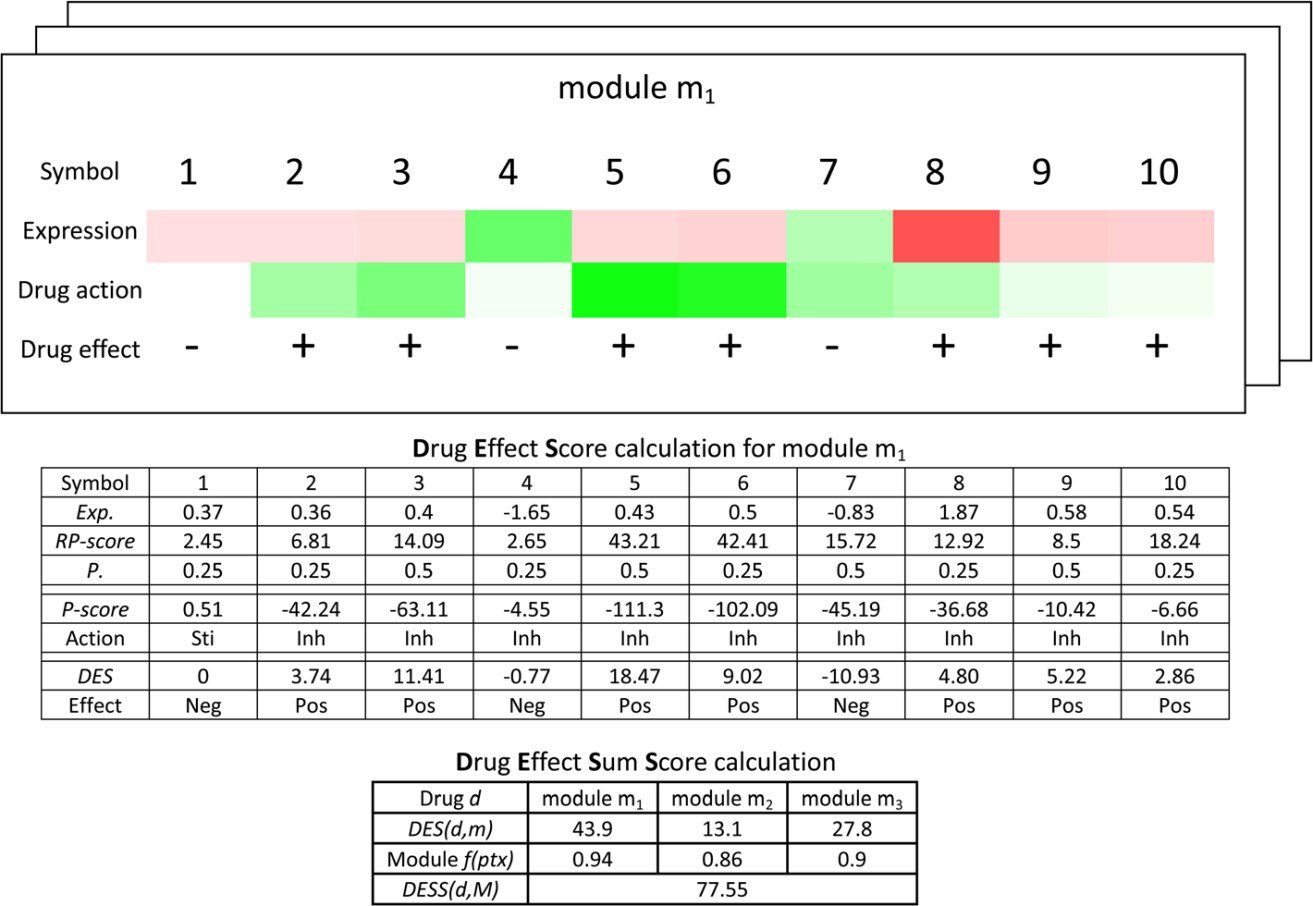
An example of calculating the *DESS* for PD-specific gene expression modules. Green indicates increased gene expression, red indicates a decrease. Note that the drug action acts to reverse the direction of gene expression found in the pathological state. *Exp.* stands for expression value, *RP-score* stands for the protein relevant score, *p.* stands for the priority score, *P-score* stands for the intuitive pharmacology score, *DES* stands for the Drug Effect Score, module *f(ptx)* stands for the enrichment score, and the *DESS* stands for Drug Effect Sum Score

The *DESS(d,M)* was calculated by integrating all PD-specific modules according to the expression:

7 $$ DESS\left(d,M\right)={\sum}_{m\epsilon Module}^M DES\left(d,m\right)\times {f}_m(pts) $$

where *M* is the module set of module *m*, *f*_*m*_(*pts*) is probability mass function (*pmf*) transform score of the PD-specific module *m*. An example of how the *DESS* score is calculated for a drug is shown in Fig. 2. Based on the total *DESS*, modular drugs (drugs selected based on their predicted effect at a module level) and their targets in the modules were collected. We pulled out modular drugs or drugs selected based on their predicted effect at a module level alongside their associated targets. Finally, we applied a single regulatory layer expansion and retrieved drug-target regulatory relationships (DMAP database) and protein-protein interactions (HAPPI-2 database) to generate the “extended modular drug-target network”.

## Results

### Construction of PD genetic association networks

**Fig. 3:**
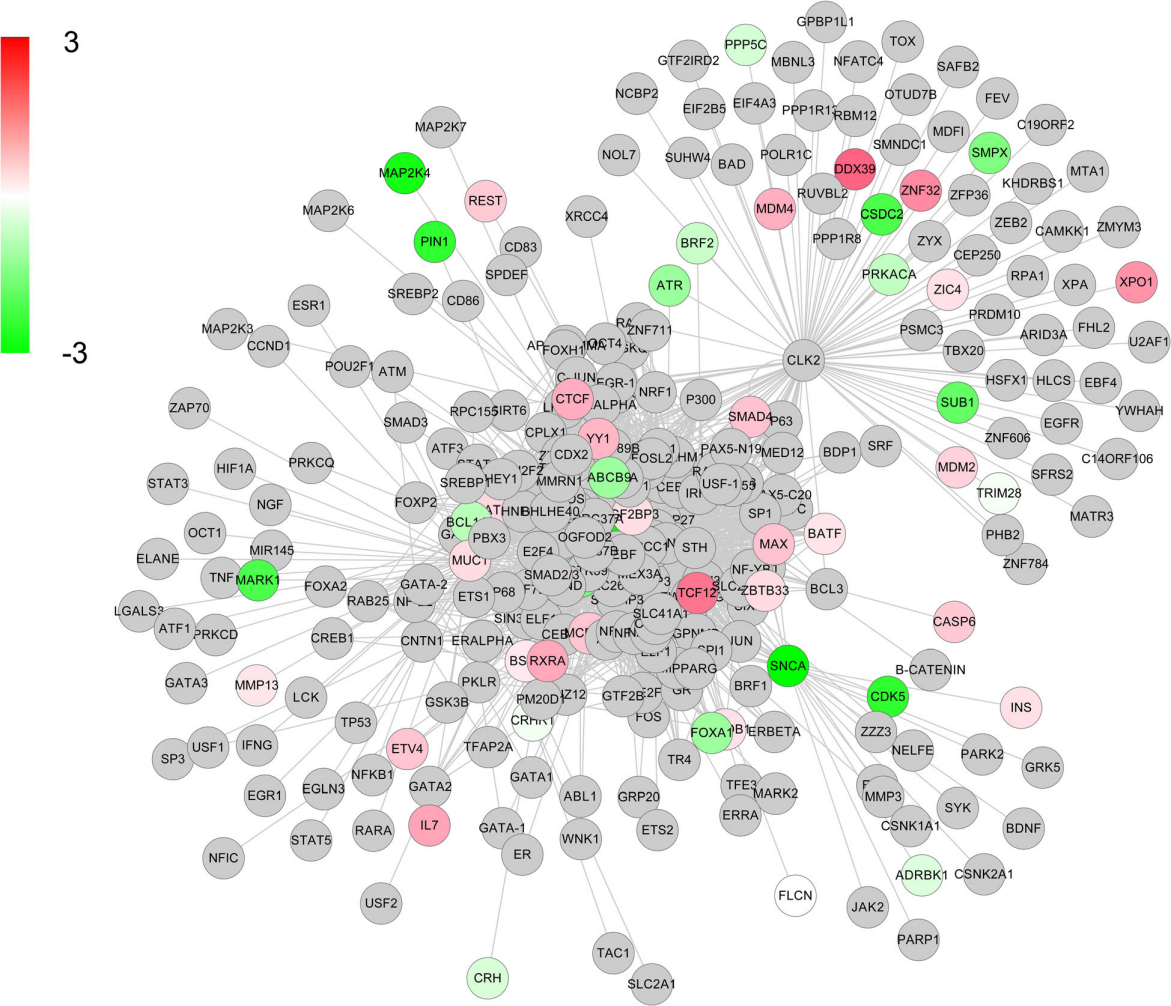
The expanded regulatory relational network generated. The color of the nodes indicates the direction of change of expression; red nodes indicate the up-regulated genes, while green nodes stand for the down-regulated genes. Nodes in gray were not assayed by our whole-genome transcriptional profiling. The color scale measures the expression changes accumulated from the three brain regions

The PD genetic association network was constructed using the neighborhood extension method. Starting from the original 54 genes identified using GWAS (described in Materials and Methods, above), we obtained PD genetic association networks consisting of a total of 288 genes and 1983 regulatory relationships. The candidates of significant expression change (eBayes moderates t-test *p-value* ≤ 0.05) are colored in the PD genetic association networks provided in Fig. 3.

### PD-specific network modules identified

**Fig. 4:**
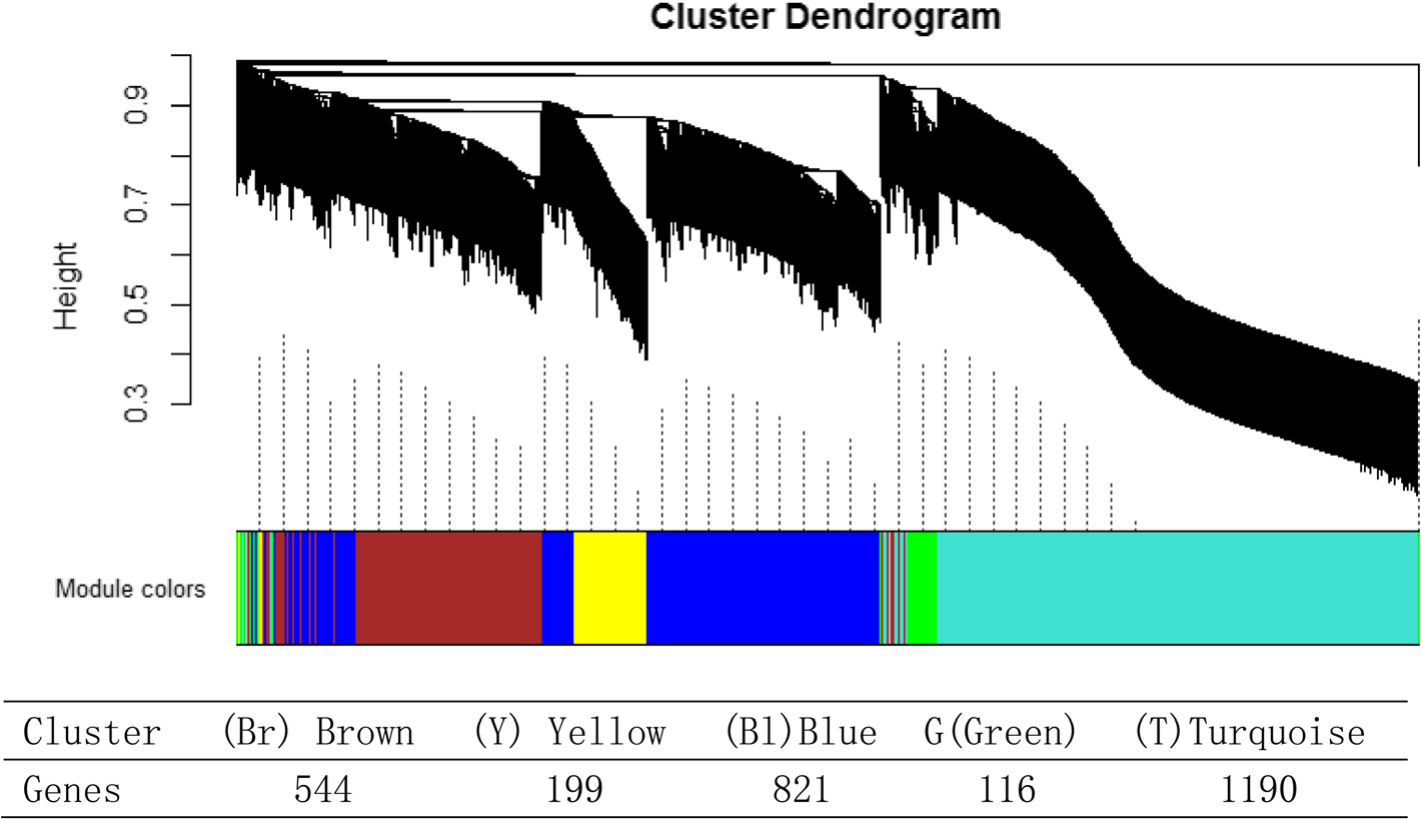
The WGCNA analysis of the five co-expression modules - Brown (Br), Yellow (Y), Blue (Bl), Green (G), and Turquoise (T). The dendrogram illustrates the degree similarity using hierarchy tree of TOM-based dissimilarity distance in each module cluster, which forms the basis for subsequent functional pathway identification

The details of the gene co-expression network construction with WGCNA have been previously described [13]. By applying the steps described above in Materials and Methods, 5 co-expression modules were identified. We color-coded these as the **Br**own (**Br**) module, the **Y**ellow (**Y**) module, the **Bl**ue (**Bl**) module, the **G**reen (**G**) module and the **T**urquoise (**T**) module, all of which are shown in Fig. 4. The number of genes in each module is as follows: the Br module containing 544 genes, the Y module containing 199 genes, the Bl module containing 821 genes, the G module containing 116 genes, and the Turquoise containing 1190 genes.

### Enrichment analysis results of two PD-specific network modules

**Fig. 5:**
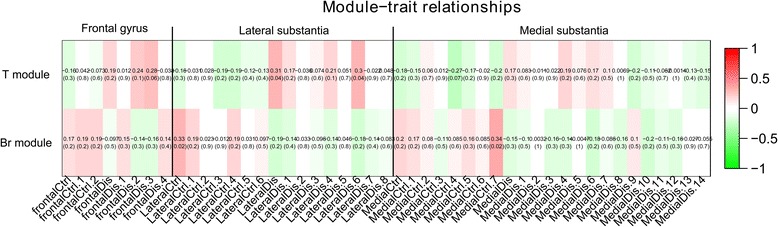
Phenotypes corresponding to each module. The color scale indicates the Pearson correlation between the samples and the modules. The number in the brackets indicates the asymptotic *P*-value for each correlation. In sample names, the “Ctrl” indicates control samples and “Dis” indicates the disease samples. The direction of the correlation differs for case and control samples in each brain region, demonstrating that the gene modules differentiate them well. In student t-test, T module frontal gyrus case VS control is 0.06, latera; substantia case VS control is 6.3×10^−4^, medial substantia case VS control is 0.03, Br module frontal gyrus case VS control is 0.04, latera; substantia case VS control is 2.2×10^−3^, medial substantia case VS control is 1.2×10^−3^

Based on the enrichment analysis, we identified two PD-specific modules (the T module and the Br module) shown in Table 1 and Fig. 4. The genes in these modules as displayed in the dendrogram are grouped tightly enough to be susceptible to a modular drug (a drug that acts on many members of the PD-specific module rather than on one target). 2895 genes are included in the gene co-expression modules. 50 of these genes in the co-expression modules overlapped with genes identified from our analysis of genetic data. These 50 genes are distributed among the modules as follows: 21 in the T module, 10 in the Br module, 2 in the G module, 3 in the Y module, and 13 in the Bl module. Using the hypergeometric test, we identified two PD-specific modules (modules having positive *f(pts)*, see Methods) the T module, which had *f(pts)* = 0.94 and the Br module, which had *f(pts)* = 0.86. Figure 5 illustrates the correlation of gene expression to case-control status. Specifically, the Pearson correlation coefficient for the expression level of the genes belonging to each module was reported for each sample. Overall, cases and controls are well discriminated by the gene expression signature of the genes in the module. For instance, in the Br module, control samples have a positive correlation with modular gene expression, while disease samples are negatively correlated with gene expression of genes found in each module. The remaining relationships are illustrated in Fig. 5. There are 2 comparisons (lateral control VS lateral case in both T-module and Br module) significantly different with *p-value* ≤ 0.001 in the student t-test.

**Table 1:** The 5 co-expression module enrichment based on GWAS results

Module	Genes in all	Candidate	Module	Module candidate	*f(pts)*	Rank
Turquoise	2895	50	1190	21	0.94	1
Brown	2895	50	544	10	0.86	2
Green	2895	50	116	2	−0.55	3
Yellow	2895	50	199	3	−0.65	4
Blue	2895	50	821	13	−0.92	5

**Fig. 6:**
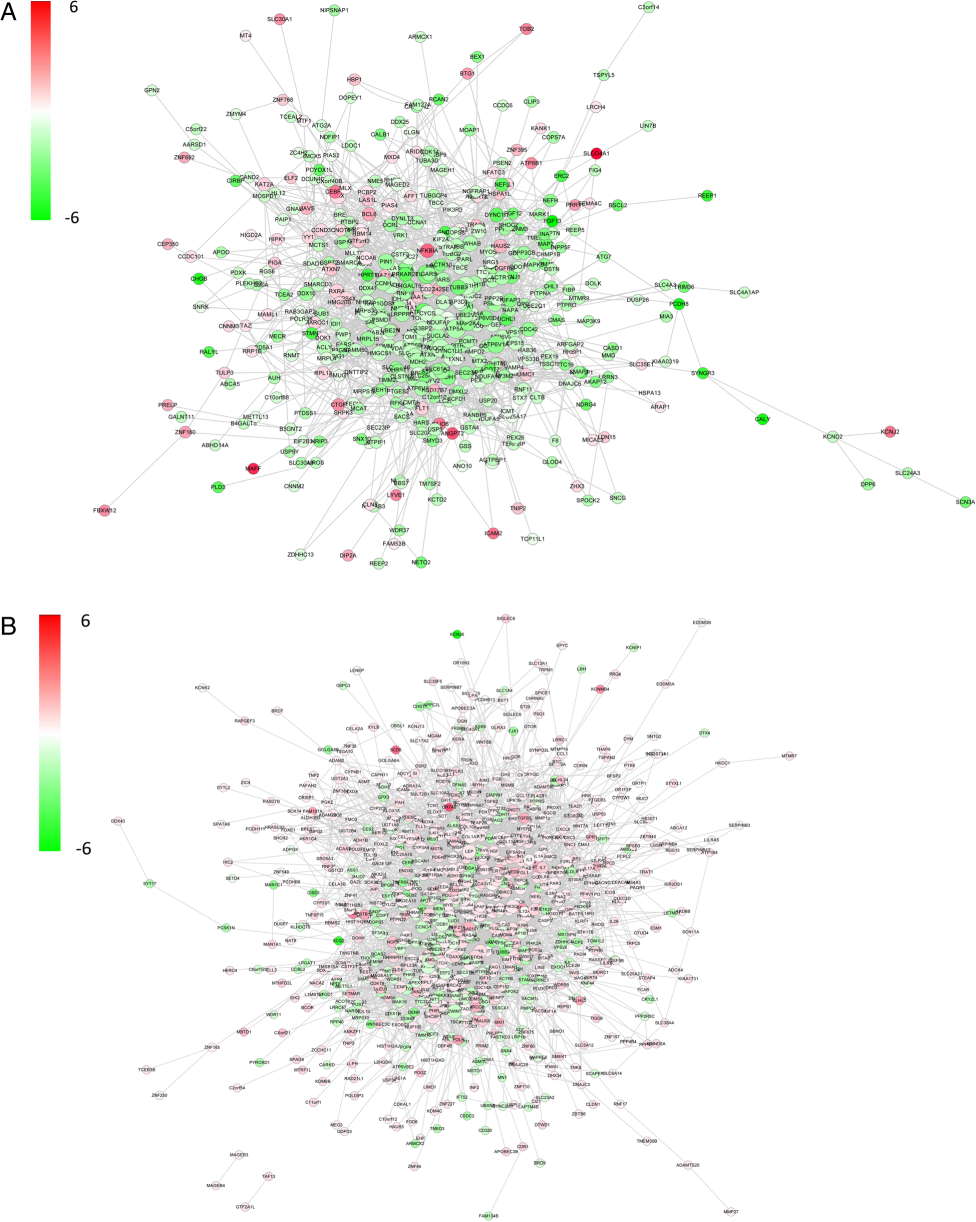
Global view of the protein-protein interaction network of the 2 modules. **a**. The Br module consists of 449 genes and 2373 gene-gene interactions. **b**. The T module consists of 905 genes and 5156 gene-gene interactions. The nodes in red color are up-regulated and the nodes in green color are down-regulated

The Br network module (Fig. 6) contains 449 genes and 2373 gene-gene interactions, of which 94 genes are up-regulated and 355 genes are down-regulated. The T network module contains 905 genes and 5156 gene-gene interactions, of which 221 genes are down-regulated and 684 genes are up-regulated.

### ClueGO analysis of PD-specific modules

**Fig. 7:**
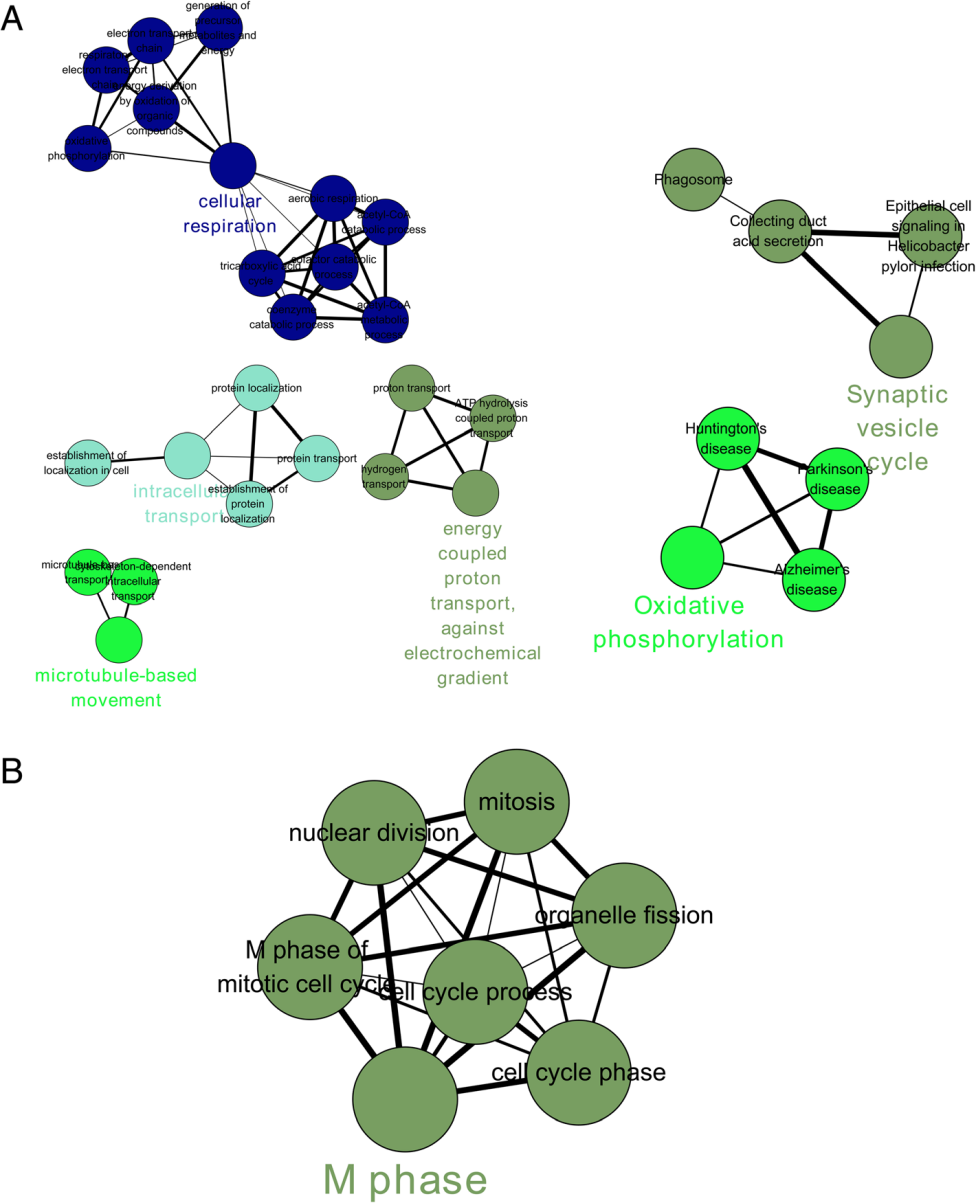
Gene Ontology - biological processes (GO-BP) relating to each PD-specific module as identified by ClueGO analysis. **a**. The GO-BP and KEGG pathways associated with the Br module. **b**. The GO-BP associated with the T module

The ClueGO analysis of the Br modules identified 4 GO biological processes, which are shown in Fig. 7 and Table 2. These are cellular respiration, intracellular transport, energy-coupled proton transport, and microtubule-based movement. Furthermore, we identified two KEGG [21] pathways, “synaptic vesicle cycling” and “oxidative phosphorylation”. The ClueGO analysis of the T module identified one GO biological process “M phase”.

**Table 2:** Gene Ontology - biological processes (GO-BP) relating to the two PD-specific modules

Module	Function	Groups	Gene numbers
Br module KEGG pathway	Oxidative phosphorylation	Group1	25
	Synaptic vesicle cycle	Group0	23
Br module GO-BP	cellular respiration	Group3	35
	energy coupled proton transport, against electrochemical gradient	Group0	10
	intracellular transport	Group2	104
	microtubule-based movement	Group1	17
T module GO-BP	M phase	Group0	94

### Identifying drugs with predicted therapeutic effects on the Br and T modules

**Fig. 8:**
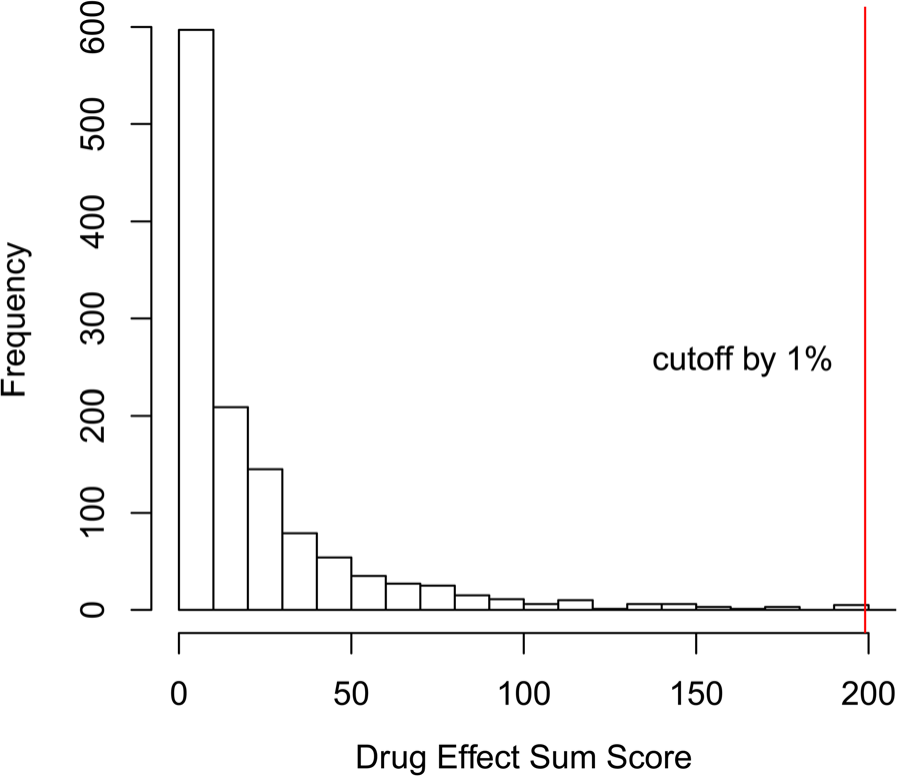
Distribution of Drug Effect Sum Score (*DESS*). The top 1% of the drugs were validated using the literature. The red line indicates the cutoff value of the *DESS* 1% drugs

**Fig. 9:**
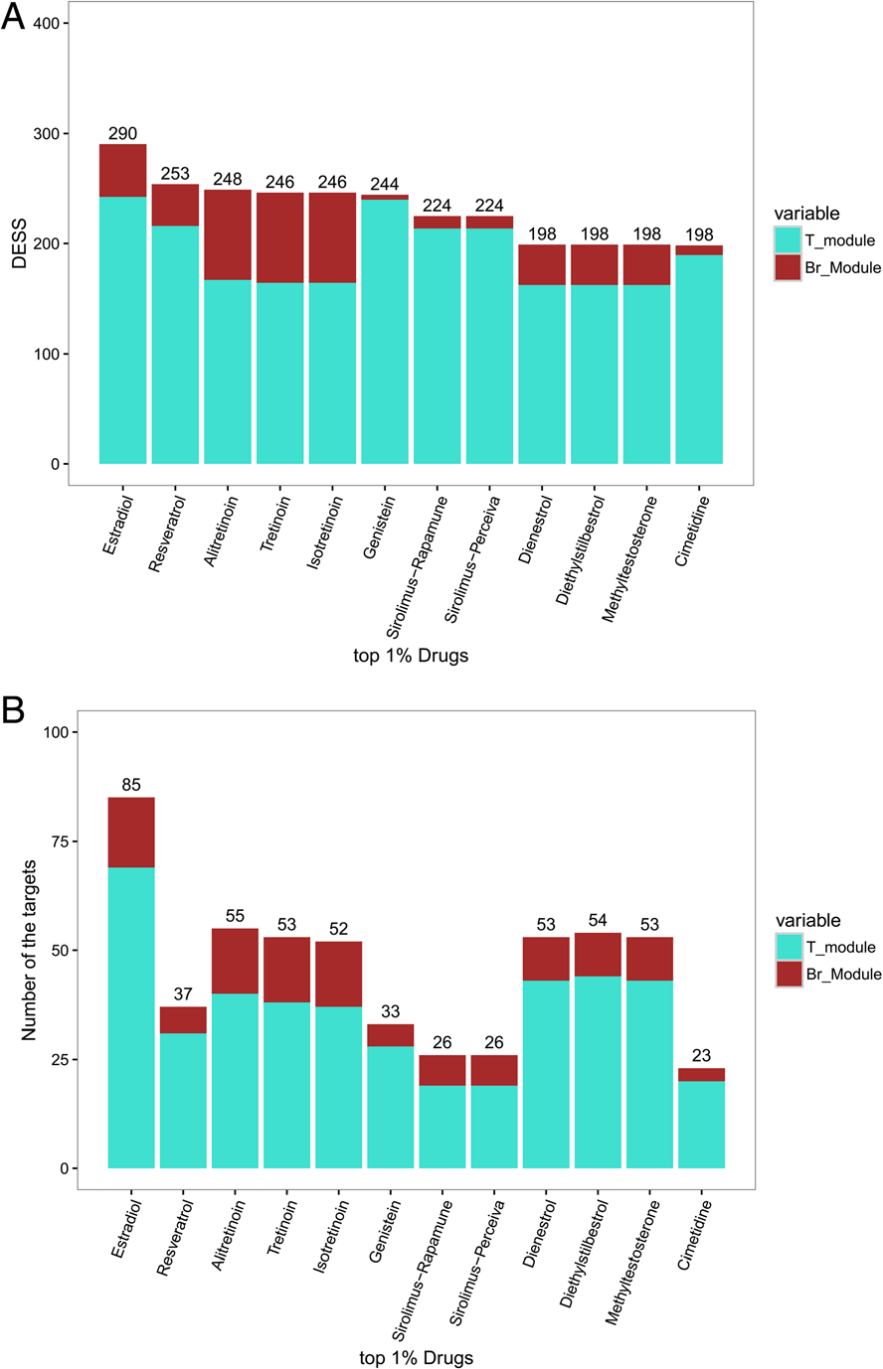
Stacked bar graph of *DESS* for highly ranked modular drugs. **a**. The top 12 most highly ranked modular drugs by *DESS*. The height of each T module and Br module stack corresponds to the Drug Effect Score (*DES*) score therapeutically modulated by each agent. **b**. The number of genes targeted by the most highly ranked modular drugs. The height of each T module and Br module stack corresponds to the drug targets therapeutically modulated by each agent

**Fig. 10:**
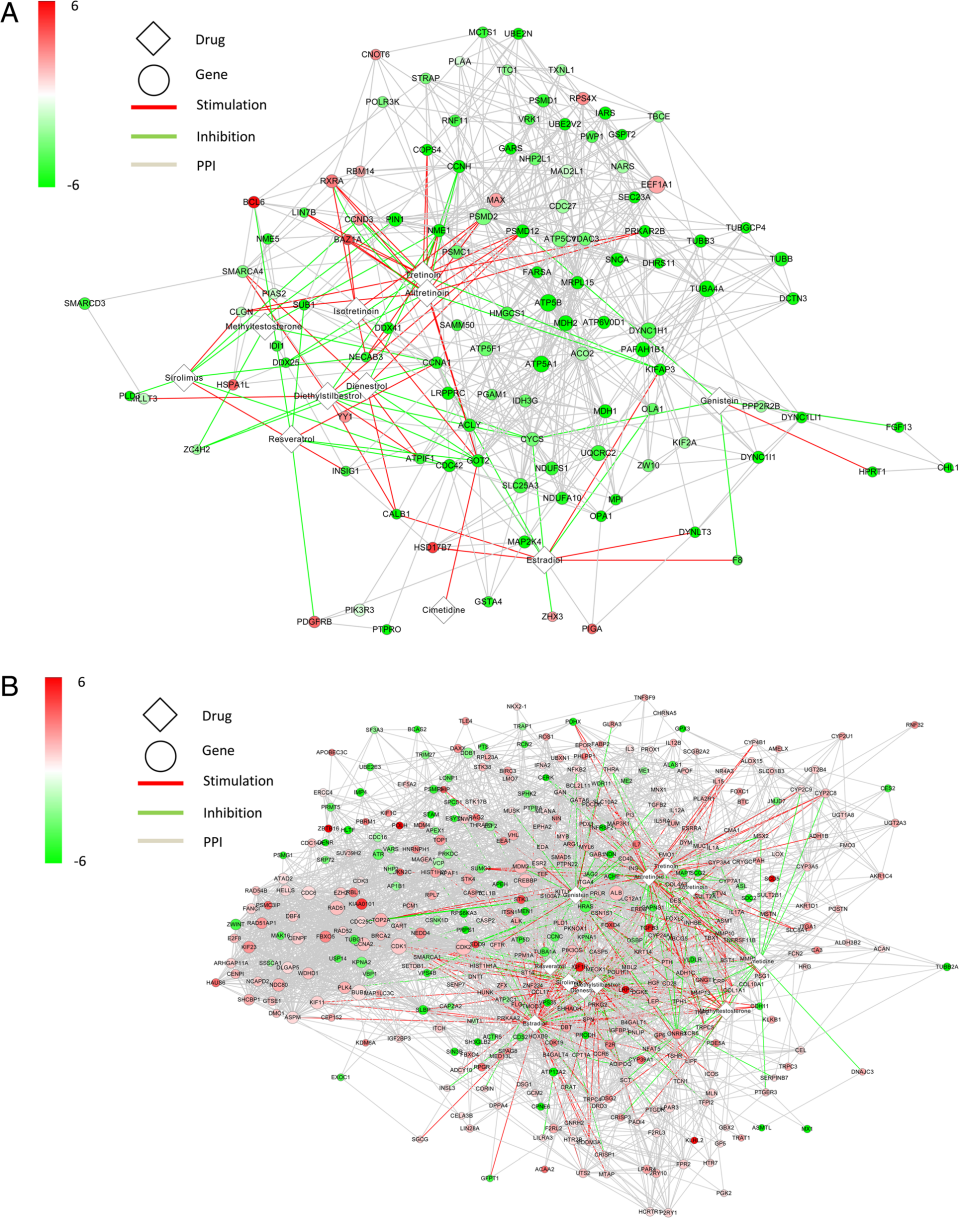
The extended modular drug-target network for the Br module (**a**) and the T module (**b**). The diamond nodes are drugs; circles are genes. The color of the nodes varies from green to red and indicates down-regulation or up-regulation situation of disordered genes, respectively. The color of the edges stands for the type of action. Red edges mean stimulation, green edges mean inhibition and gray edges means Protein-Protein Interactions. The node size represents the RP-score which indicates the relevance of the gene in the module calculated in Method

We generated a ranked list of the drugs based on their *DESS* scores. While there were 1246 (1201 unique drugbankID) candidate drugs for drug repositioning that targeted one or more genes in the gene co-expression module in Additional file 1: Table S1, we selected only 12 (the top 1% according to *DESS*) candidate drugs as potential treatments (Fig. 8). The components of *DESS* and number of the drug targets for each drug in the T module and Br module are shown in Fig. 9. Furthermore, the drugs are listed in Table 3 and are discussed below. The Br and T modules’ network diagrams for the extended network illustrating which disordered genes are stimulated and inhibited by these 12 drugs is provided in Fig. 10, Additional file 2: Table S2 and Additional file 3: Table S3.

**Table 3:** Compound IDs for the 12 most highly ranked modular drug candidates

Compound ID	Compound Name	Score	Drugbank	DrugName	Class
CID5757	CID5757	290.3	DB00783	Estradiol	Steroids and steroid derivatives
CID445154	SAM001246888	253.9	DB02709	Resveratrol	Stilbenes
CID444795	Retinoic Acid	248.9	DB00523	Alitretinoin	Prenol lipids
CID5538	Accutane Roche	246.3	DB00755	Tretinoin	Lipids and lipid-like molecules
CID5282379	Isotretinoin (USP)	246.3	DB00982	Isotretinoin	Prenol lipids
CID5280961	NCGC00025005–02	244.3	DB01645	Genistein	Isoflavonoids
CID5460439	Rapamune	224.9	DB00877	Sirolimus	Macrolide lactams
CID6436030	Perceiva	224.9	DB00877	Sirolimus	Macrolide lactams
CID667476	follidiene	199	DB00890	Dienestrol	Phenylpropanoids and polyketides
CID448537	oekolp	199	DB00255	Diethylstilbestrol	Phenylpropanoids and polyketides
CID6010	component of Tylosterone	199	DB06710	Methyltestosterone	Lipids and lipid-like molecules
CID2756	C4522_SIGMA	198.2	DB00501	Cimetidine	Organoheterocyclic compounds

## Conclusions and discussion

In this work, we present a framework that identified candidate drugs for repositioning based on analysis of GWAS and gene expression microarray data. Starting with genes identified through a standard GWAS, we extended the analysis to one-layer extension by gene-gene regulatory relationship and built an extended regulatory network. Significant results based on an enrichment analysis were then used to generate PD modules. We improved gene co-expression module cohesion by removing isolated or weakly connected genes. PD network modules were then further informed by the integration of data from Protein-Protein interaction databases.

Using this approach, we initially identified over 1201 candidates for drug repurposing. We trimmed this to 12 modular drug candidates based on their *DESS*. There were three important characteristics of finding within these 12 modular drugs. First, they are noteworthy in that they target PD at the level of the gene co-expression module as opposed to a specific target. Second, most of the genes on the list belong to drug families, which should be expected if data relating to drug target efficacy are accurate and internally consistent. Third, there are general 4 drug families found (steroids and steroid derivatives, lipids and lipid-like molecules, phenylpropanoids and polyketides, and other small molecules), and each family of drugs identified has been previously studied in relation to neurodegenerative disease, suggesting the external validity of our findings as well.

The top candidate drug was estradiol, a steroidal estrogen critical in the regulation of the menstrual cycle. It is currently used pharmaceutically in hormone replacement therapies for menopause and hypogonadism. Several studies support a role for the use of estradiol in PD. It has been shown to protect dopaminergic neurons in an MPP+ Parkinson’s disease model [22], and a study of postmenopausal women found it to be associated with a reduced risk of PD in women [23]. Further, it is well-established that estrogen deprivation leads to the death of dopaminergic neurons. Of note, many clinical reports also suggest an anti-dopaminergic effect of estrogens on symptoms of PD. It is likely that the timing and dosage of estrogen influence the results in these discrepant findings. Our ninth, tenth and eleventh-ranked drugs (dienestrol, diethylstilbestrol, and methyltestosterone respectively) are isomers relating to diethylstilbestrol (also known as follidiene). Diethylstilbestrol is a synthetic non-steroidal estrogen previously used to treat menopausal and postmenopausal disorders. However, it is now known to have teratogenic and carcinogenic properties [24]. Although these compounds may be contraindicated for use in humans, their high prioritization might prompt us to look for similar compounds without carcinogenic side effects. Methyltestosterone, which had the tenth highest *DESS*, is a synthetic orally active androgenic-anabolic steroid with relatively high estrogenicity. Methyltestosterone is currently used to treat males with androgen deficiency. Interestingly, testosterone deficiency has previously been reported in patients with PD, and PD itself is seen more commonly in men than women [25]. However, clinical trials have shown no improvement in male PD patients when given exogenous testosterone therapy [26]. Finally, our sixth most highly ranked drug was genistein, an estrogen-like isoflavone compound found exclusively in legumes. Genistein is known to act as an angiogenesis inhibitor and was previously shown to have neuroprotective effects on dopaminergic neurons in mouse models of PD [27].

Resveratrol had the second highest DESS. It is a polyphenolic anti-oxidant stilbenoid compound found in food include the skin of grapes, blueberries, raspberries and mulberries, currently under preclinical investigation as a potential pharmaceutical treatment in treating early onset PD patients. Resveratrol was previously studied in a phase-II clinical trial for individuals with mild to moderate Alzheimer’s disease and was found to reduce plasma levels of some AD biomarkers [28–30].

The third drug alitretinoin, fourth drug tretinoin, and fifth drug isotretinoin are most highly ranked candidates also belonging to a single family of compounds, retinoids. The first is retinoic acid, a retinoid morphogen crucial to the embryonic development of the anterior-posterior axis in vertebrates, as well as differentiation and maintenance of neural cell lineage. Currently, in-vivo animal studies suggest the possibility of therapeutic applications of retinoic acid for PD through nanoparticle delivery [31]. Isotretinoin, trademarked under the name Accutane, is prescribed as a treatment for severe acne vulgaris. Although isotretinoin is a known teratogen [32], it might be well-suited to treatment of PD given its typical later age of onset.

Our seventh and eighth hits, Sirolimus (Rapamune) and Sirolimus (Perceiva), are again related. Perceiva is an ocular formulation of the macrolide compound sirolimus (commonly known as rapamycin) and was developed to treat neovascular age-related macular degeneration. Sirolimus is used for the treatment of Lymphangioleiomyomatosis, as well as in prevention of organ transplant rejection. Interestingly, sirolimus has been shown to improve cognitive deficits in mouse model of Alzheimer’s Diseases through inhibition of the mTOR signaling pathway, a pathway which is thought to protect against neuronal death in mouse models of PD [33].

In addition to these twelve candidates, our ClueGO analysis suggests that investigation of two additional biological processes may be profitable. Our analysis of KEGG pathways in relation to the T module implicated mitochondrial respiration as a potential disease mechanism [34]. Interestingly, it has previously been reported that defects in mitochondrial respiration are etiologically related to the pathogenesis of PD. Thus, preservation and restoration of mitochondrial function may hold promise as a potential therapeutic intervention to halt the progression of dopaminergic neurodegeneration in PD. Secondly, in PD, neuronal cells undergo mitotic catastrophe and endoreduplication prior to cell death. It has previously been shown [35] that overexpression of DNA poly β was involved in the rotenone-mediated pathology of cellular and animal models of PD. In a cell culture model, increased levels of DNA poly β promoted rotenone-mediated endoreduplication. Selective injury to dopaminergic neurons by rotenone resulted in the upregulation of DNA poly β as the neuronal cell cycle was reactivated.

In summary, we perform drug repositioning by integrating weighted drug-protein regulations on all genes, using our novel *DESS* to quantitate drug effects on entire co-expression networks. As biological systems use functional pathways and networks to maintain homeostasis, by selecting drugs that act at the level of a gene module we were able to target a level of the biological organization more relevant to the disease pathologies of complex disorders such as PD. Although this approach is still in its infancy, our results suggest that it may circumvent issues associated with single-gene targeting in polygenic diseases like PD. Our analysis has identified several families of related drug candidates, all of which have previously been investigated in relation to PD and other neurodegenerative diseases. As such, we believe our framework gives internally and externally valid results and may be extended to provide complementary insights to other disease-module findings and drug-repositioning projects.

The significance of our work should be considered in light of its limitations. First, several of the classes of drugs mentioned have already studied in relation to PD and related phenotypes, as described above. However, members of the families of drugs identified have not resulted in a clinically efficacious treatment for PD to date. As such, a future direction for this line of research is to include a mechanism to account for both additive and potentially non-additive interaction effects between drugs on a disease-specific module. In addition, many of the most highly ranked modular drugs we identified show much promise, but have known adverse effects. Future research will include a method of incorporation of drug side effects into the final priority score.

## Additional files

Additional file 1: 1246 (1201 unique drugbankID) candidate drugs for drug repositioning that targeted to PD modules. (XLSX 136 kb)

Additional file 2: The Protein-Protein Interactions and drug-target regulations in Br module’s network. (XLSX 26 kb)

Additional file 3: The Protein-Protein Interactions and drug-target regulations in T module’s network. (XLSX 98 kb)
